# Pyruvate Plays a Main Role in the Antitumoral Selectivity of Cold Atmospheric Plasma in Osteosarcoma

**DOI:** 10.1038/s41598-019-47128-1

**Published:** 2019-07-23

**Authors:** Juan Tornin, Miguel Mateu-Sanz, Aida Rodríguez, Cédric Labay, Rene Rodríguez, Cristina Canal

**Affiliations:** 1 Biomaterials, Biomechanics and Tissue Engineering Group, Dpt. Materials Science and Metallurgy, Technical University of Catalonia (UPC), Escola d’Enginyeria Barcelona Est (EEBE), c/Eduard Maristany 14, 08019 Barcelona, Spain; 2 Barcelona Research Center in Multiscale Science and Engineering, UPC, Barcelona, Spain; 3 Hospital Universitario Central de Asturias - Instituto de Investigación Sanitaria del Principado de Asturias, Av. de Roma s/n, Oviedo, Spain; 4 Instituto Universitario de Oncología del Principado de Asturias, Av. de Roma s/n, Oviedo, Spain; 5 CIBER oncology (CIBERONC), Madrid, Spain

**Keywords:** Bone cancer, Plasma physics, Medicinal chemistry

## Abstract

Osteosarcoma (OS) is the most common primary bone tumor but current therapies still have poor prognosis. Cold Atmospheric Plasma (CAP) and Plasma activated media (PAM) have shown potential to eliminate cancer cells in other tumors. It is thought that Reactive Oxygen and Nitrogen species (RONS) in PAM are key players but cell culture media composition alters treatment outcomes and data interpretation due to scavenging of certain RONS. In this work, an atmospheric pressure plasma jet was employed to obtain PAM in the presence or absence of pyruvate and used to treat the SaOS-2 (OS) cell line or hBM-MSC healthy cells. OS cells show higher sensitivity to PAM treatment than healthy cells, both in medium with and without pyruvate, activating apoptosis, DNA damage and deregulating cellular pathways mediated by c-JUN, AKT, AMPK or STAT3. In line with previous works, lack of pyruvate increases cytotoxic potential of PAM affecting cancer and healthy cells by increasing 10–100 times the concentration of H_2_O_2_ without altering that of nitrites and thus decreasing CAP anti-tumor selectivity. Suitable conditions for CAP anti-cancer selectivity can be obtained by modifying plasma process parameters (distance, flow, treatment time) to obtain adequate balance of the different RONS in cell culture media.

## Introduction

Osteosarcoma is the most common primary bone tumor and accounts for approximately 19% of all malignant tumors in bone and 40–60% of all primary malignant tumors of bone^1,2^. Despite being rare, it is the most common solid tumor in teenagers and the third most common malignancy in children, accounting for 7.5% of all adolescent cancers^3^. Because of the effects of radical surgery and chemotherapy, a significant reduction in the quality of life is found in children and adolescents. Moreover, for the last 20 years the 5-year survival has plateaued at approximately 70% and long-term complications of osteosarcoma survivors treated with intensive chemotherapy have increased^4^. With the promising perspective of a new oncological therapy, cold atmospheric plasmas and plasma-activated liquids have come to the limelight. Cold atmospheric plasma (CAP) is an ionized gas at near-room temperature composed of a high number of reactive species, ions, electrons, metastable particles, electromagnetic field, and weak UV and VIS radiation. These plasma-generated reactive oxygen and nitrogen species (RONS), can be transferred to liquids through secondary reactions. Plasma-activated liquids display different biological actions which have been mainly attributed to the generation of RONS such as H_2_O_2_ ^5,6^, NO_2_^–^ ^7^, ONOO^−^ ^8^, etc. These reactive species are known to be involved in a wide range of intracellular^6,7,9^ and intercellular processes^10^ and have shown a strong anti-cancer effect on several cancer cell lines *in vitro*^11–17^ and on subcutaneously xenografted tumors *in vivo*^14,17–21^. Recently, proof of efficiency and selectivity in osteosarcoma was shown^10,22–24^. However, the exact mechanisms in OS remain to be elucidated. In other cancer types, it has been reported that CAP treatment induces an increase of intracellular ROS (reactive oxygen species) in cancer cells^20,25^ associated to failures in antioxidant defenses^26,27^, DNA damage^28,29^, cell cycle arrest^30,31^ and apoptotic^32^ or necrotic^33^ cell death. Simultaneously, healthy cells show stronger resistance to CAP treatment^34^.

Besides, the effects of different plasma-activated liquids, including cell culture media, have been evaluated^35^. Following plasma treatment, these liquids are known as Plasma Activated Media (PAM) and have been shown to have cytotoxic effects on cancer cell cultures. As mentioned, one of the advantages commonly attributed to CAP is its antitumoral selectivity, without affecting healthy cells. CAP efficiency is based on the concentration and kind of RONS generated^36,37^. Exhaustive determination of all RONS present in PAM is far from straightforward, and the most often quantified species are hydrogen peroxide, nitrites and nitrates. It is known, however, that a much more complex cocktail of species is formed in PAM through diffusion from the gas phase to the liquid. It is claimed that this complex cocktail of RONS is the main responsible for the CAP-induced anticancer action^38–40^. The concentration and type of RONS depend enormously on plasma application parameters, such as distance or treatment time^41^, as well as on the chemical/biochemical composition of the liquid^39,42^.

So far, only a few works have addressed the differential effects of cold plasma on tumor and non-tumor cells using the same experimental settings^24,36,40,43–46^. In addition, previous reports tend to exclude sodium pyruvate (Pyr) from culture media formulations as it seems that this specific scavenger of H_2_O_2_ may reduce the efficiency of the plasma treatment *in vitro*^25,47^. However, Pyr is a key player on cancer metabolism and is commonly found in most cell culture media so this absence in CAP-related works deserves investigation. Adachi *et al*., showed that cell culture media composition determines PAM cytotoxic potential on cancer cells, and specifically Pyr reduces cell damage on cancer cells^48^. In other studies, Pyr has been successfully used to eliminate ROS induced angiogenesis^49^ and proliferation^50^, by suppressing FGF2 release from plasma treated cells, suggesting that Pyr can affect intracellular and extracellular plasma effects.

Altogether, we aimed at defining the specific conditions in which CAP treatment shows a selective cytotoxic effect on tumor cells. Specifically, we focused on clarifying the role sodium pyruvate on modulating the concentration of H_2_O_2_ and the selectivity of the anticancer effects of plasma-activated liquids. Different plasma treatment conditions of PAM (distance, gas flow, treatment time) with/without pyruvate were evaluated on the cytotoxicity of PAM, and mechanistic insights were investigated by studying cell proliferation, DNA damage, cell death mechanisms and cell signaling on osteosarcoma and non-cancer cells.

## Results

### Characterization of the plasma jet

**Figure 1:**
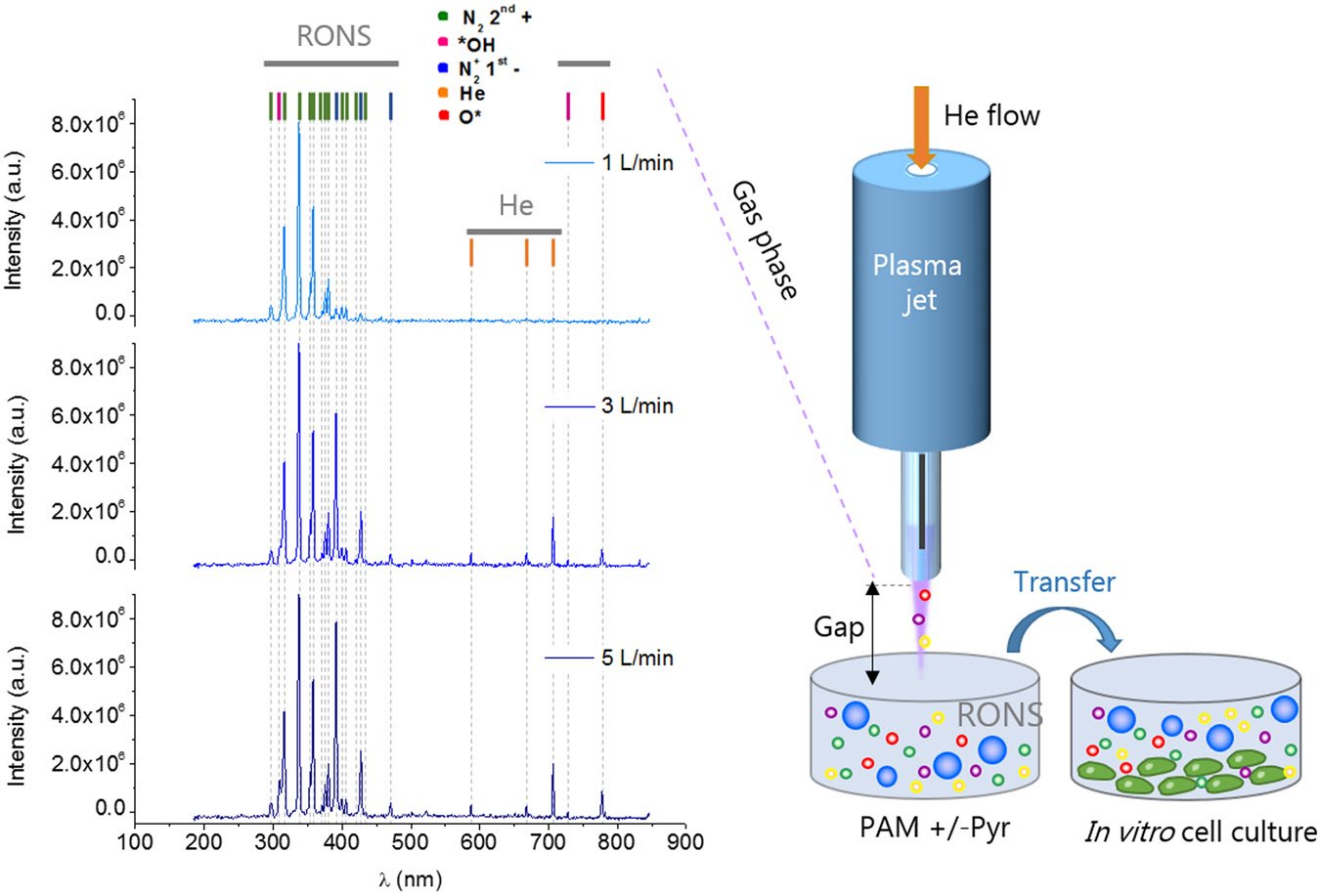
Plasma jet generates reactive species in the gas phase. Main species detected by optical emission spectroscopy in the plasma gas phase at different gas flow rates (measured at 10 mm from the exit of the jet) (Left). Schematic illustration of the generation of PAM +/− Pyr with an APPJ. Parameters such as the gap between the plasma and the liquid surface (10 or 20 mm), or the gas flow (1, 3, 5 L/min) were investigated. DMEM was treated with APPJ to obtain PAM, which is then transferred onto cell cultures.

An Atmospheric Pressure Plasma Jet (APPJ) was employed to treat cell culture media and obtain PAM (Fig. 1). Optical Emission Spectroscopy (OES) was used to analyze the species generated by plasma in gas phase (Fig. 1). OES reveals higher levels of nitrogen and oxygen radicals than excited helium atoms. The wavelengths 316, 337, 380, 706, and 777 nm were used to investigate production of *OH, N_2_ 2^nd+^, N^2+^ 1^st−^, He, and O*, respectively. Figure 1 reveals that higher gas flows (1 to 5 L/min) are accompanied by an increase in reactive species (N_2_^+^ 1st^−^, He and O*). The evolution of the peaks of the different plasma species with increasing flow rates (Table S1) showed a progressive increase of the N_2_^+^ intensity and slight increases for the rest of species.

### Sodium pyruvate attenuates PAM cytotoxicity while increasing its antitumor selectivity

**Figure 2:**
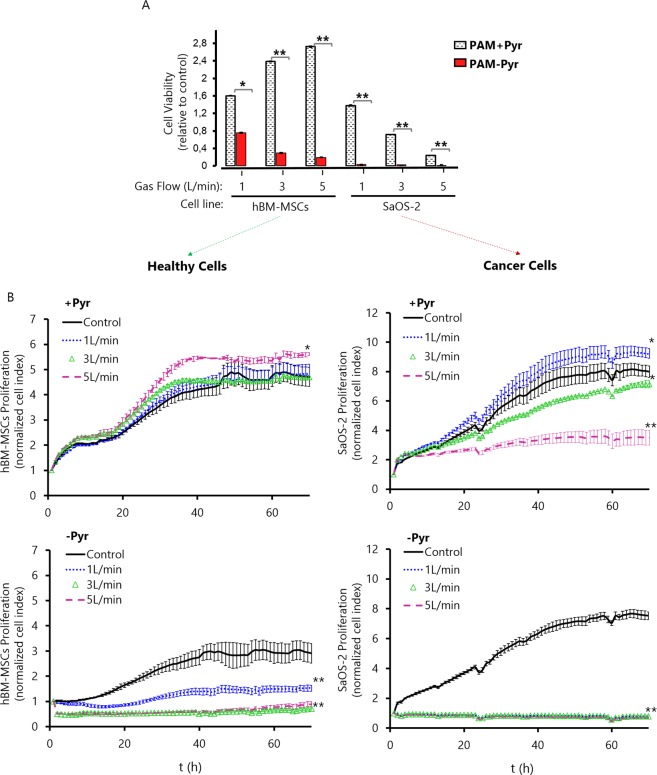
Effects of PAM on osteosarcoma and healthy cells. (**A**) Effects of PAM +/− Pyr (1, 3 and 5 L/min helium flow at 10 mm gap) on cell viability were detected by WST-1 assay on hBM-MSCs and SaOS-2, measured 24 h after exposure. Cell viability is expressed relative to the corresponding untreated control and is the mean and standard deviation of three independent experiments (*p < 0.05; **p < 0.005; two-sided Student’s t-test) (**B**) Effect of PAM +/− Pyr at different helium flows (1, 3 and 5 L/min) and 10 mm gap on the proliferation ability of hBM-MSCs healthy cells (left panel) and SaOS-2 cancer cells (right panel). We analyzed the Normalized Cell Index relative to treatment time (t = 0 h) up to final step (t = 70 h). Data are shown as the mean. Error bars represent the SD, and asterisks indicate statistically significant differences between the indicated series (*p < 0.05; **p < 0.01; two-sided Student’s t-test). Readings were done in duplicates.

To investigate the anti-tumoral selectivity of PAM, its effect on viability of osteosarcoma SaOS-2 cells and healthy human Bone Marrow Mesenchymal Stem Cells (hBM-MSCs) was evaluated (Fig. 2). In the absence of pyruvate, plasma-activated DMEM efficiently eliminated cancer cells in all conditions after 24 hours of incubation with PAM. Although more resistant than OS cells, hBM-MSCs were also sensitive to PAM-Pyr treatment in a flow-dependent fashion. On the other hand, the presence of pyruvate resulted in a strikingly different effect of PAM in healthy and cancer cells. Thus, although significantly less efficiently than in the absence of pyruvate, PAM + Pyr treatment still induced a flow-dependent cytotoxic effect in SaOS-2 cells, whereas this treatment resulted in a flow-dependent increased proliferation of hBM-MSC, rather than in a cytotoxic effect (Fig. 2A).

**Figure 3:**
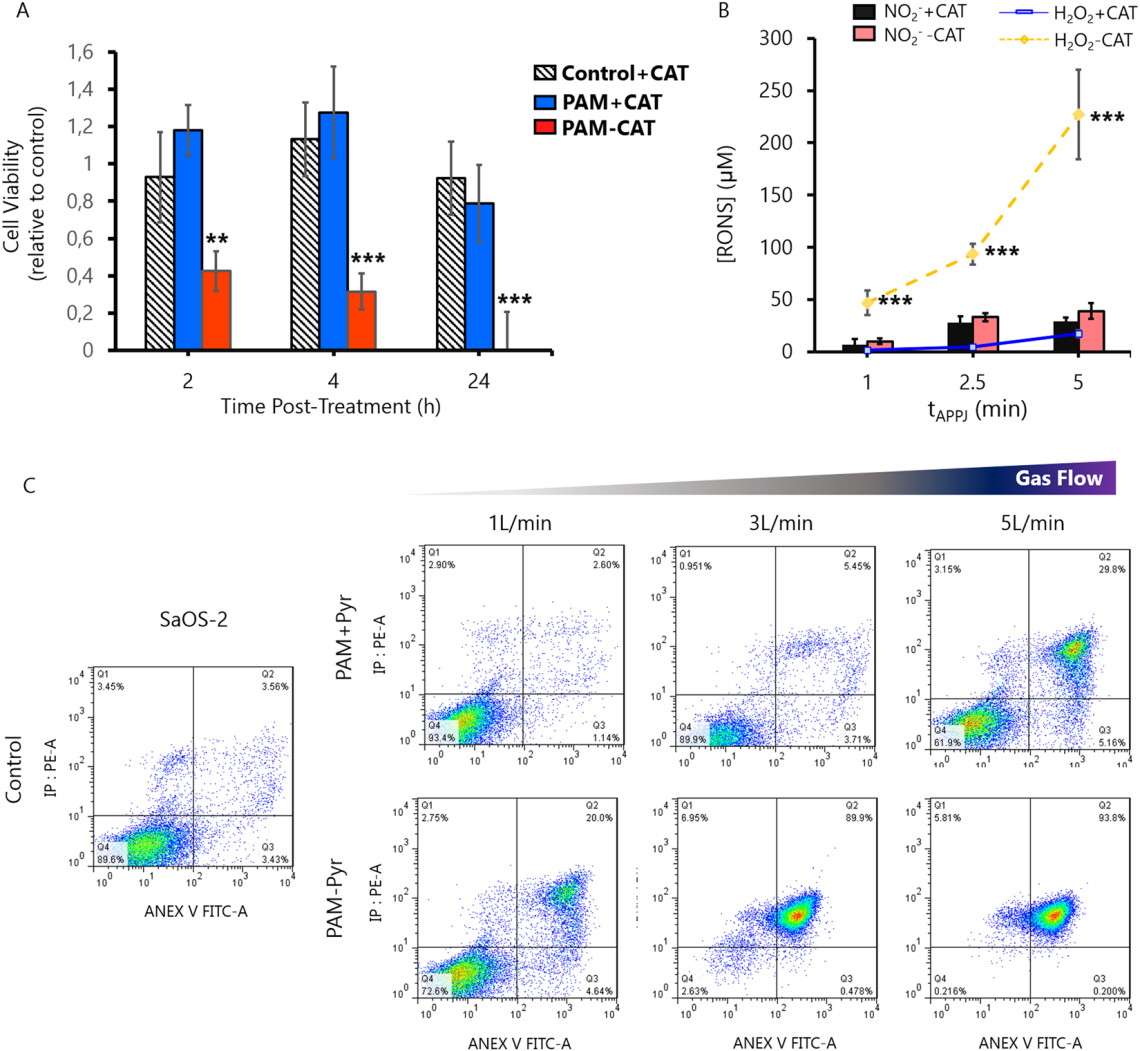
H_2_O_2_ scavengers protect osteosarcoma cells from PAM-induced apoptosis. (**A**) Effects of PAM +/− Cat activated during 5 minutes (3 L/min helium flow at 10 mm gap) were detected by WST-1 assay on SaOS-2, measured 2, 4 and 24 h after exposure. Catalase was added as control to untreated cell culture medium. Cell viability of Control + Cat, PAM + Cat and PAM-Cat is expressed relative to Control-Cat (untreated) and is the mean and standard deviation of three independent experiments (**p < 0.005; ***p < 0.001; two-sided Student’s t-test). (**B**) Concentration of NO_2_^−^ and H_2_O_2_ in PAM+/-Catalase at different treatment times (1 to 5 min at 3 L/min and 10 mm gap). Catalase addition to PAM, ^−^not lead to significant differences on the concentration of NO_2_^−^, but reduces H_2_O_2_ concentration in all conditions studied. Data are presented as mean, n = 3, Error bars represent the SD, and asterisks indicate statistically significant differences between the PAM +/− Catalase series (**p < 0.01; ***p < 0.001; two-sided Student’s t-test). Concentration of RONS was measured immediately after APPJ treatments using untreated DMEM as blank. (**C**) SaOS-2 cells were treated with PAM + Pyr (top) and PAM-Pyr (bottom) activated by plasma at different Helium flows (1, 3 or 5 L/min) during 5 minutes. Apoptosis/Necrosis activation were examined for Annexin V/PI binding using FACS analysis. PAM + Pyr at 1 L/min produces 2.6% apoptotic cells, progressively increasing with 3 L/min (5.5%) and to 5 L/min (29.8%). PAM-Pyr boosts these values, leading to 20% at 1 L/min, 89% at 3 L/min and 93% at 5 L/min of apoptosis positive cells Representative data at 24 hours are shown. Analysis were done in duplicates.

To investigate this in depth, we performed a real-time proliferation assay by monitoring cell impedance up to 70 h post-treatment (Fig. 2B). Results confirm that cancer cells are much more sensitive to PAM treatment than healthy cells. Specifically, the treatment with a flow of 5 L/min in presence of Pyruvate results in a selective elimination of OS cells without affecting healthy hBM-MSC cells. The absence of pyruvate increases the cytotoxic potential of PAM in all the studied parameters. PAM-Pyr produces a decrease of proliferation in a gas flow dependent manner, both on osteosarcoma and healthy cells. This occurs disregard the medium where cells are grown prior to contact with PAM contains or not Pyr. Thus, while the cytotoxicity is not related to lack or shock of Pyr, it is true that cells grown in DMEM-Pyr are sensitive to treatment with PAM (Fig. S1). Conversely, in osteosarcoma cells PAM -Pyr leads to 0% viability and the total suppression of the proliferative capacity at all conditions evaluated (Fig. 2A,B). To further investigate the role of plasma-generated H_2_O_2_ in cytotoxicity, catalase (Cat) was added to PAM immediately after treatment. As expected, catalase fully suppressed the cytotoxic potential of PAM in osteosarcoma cells (Fig. 3A). As observed in PAM-Pyr (Fig. 2A), PAM-Cat leads to a 70% reduction on SaOS-2 cell viability 4 h post-treatment and to 0% after 24 h. This can be attributed to the scavenging of H_2_O_2_ by catalase which does not affect the generation of NO_2_^−^ (Fig. 3B). To characterize the mechanism of cell death of SaOS-2 cells exposed to PAM +/− Pyr, activation of apoptosis or necrosis were analyzed by Annexin V/PI staining after 24 h of treatment with PAM (Fig. 3C).

**Figure 4:**
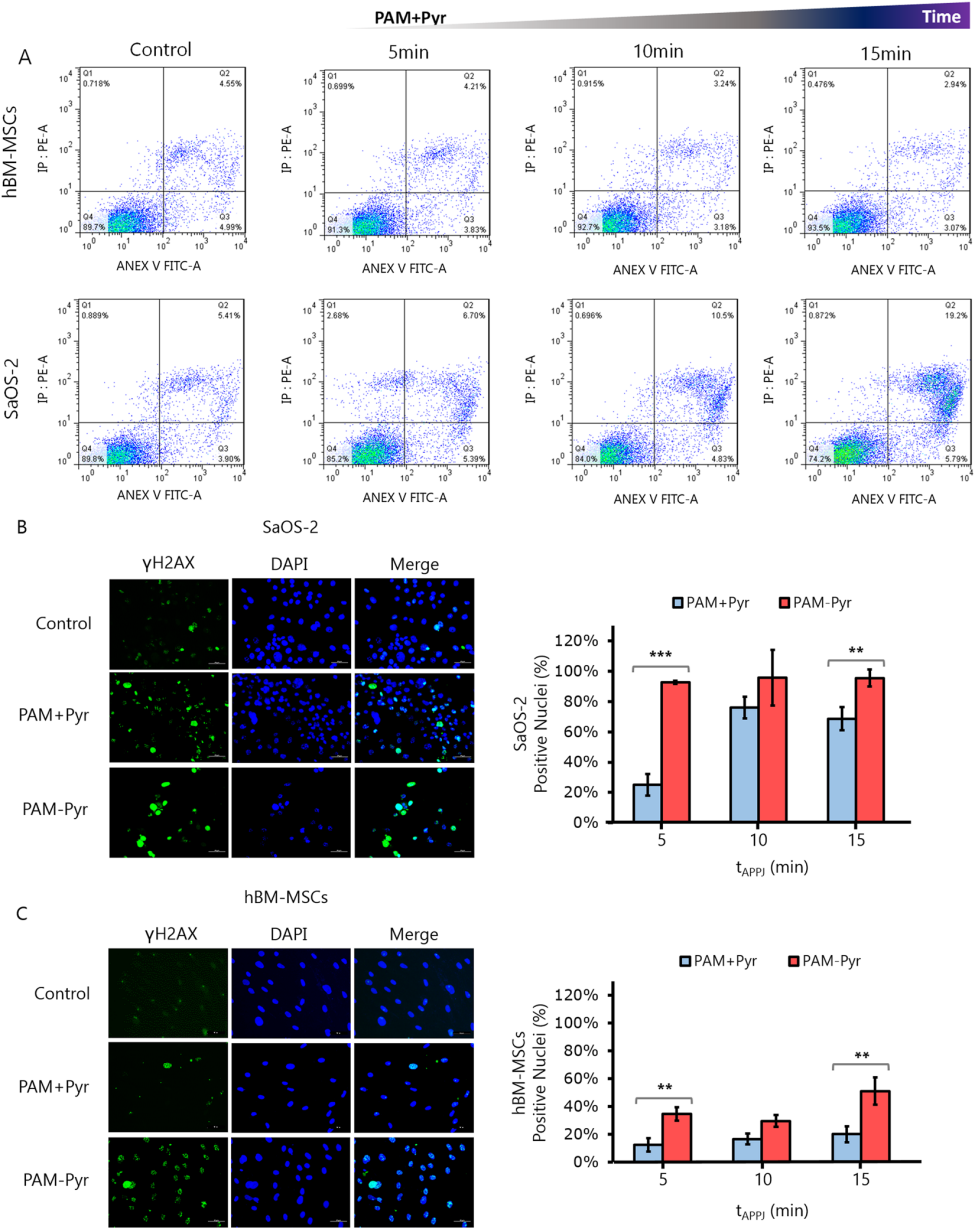
PAM induces DNA damage and apoptosis mainly in OS cells. We used different treatment times (5, 10 and 15 min) in PAM + Pyr at 1 L/min and 10 mm gap to analyze the cytoprotective effect of Pyr. (**A**) Analysis of apoptosis/necrosis by FACS using AnnexinV/PI on hBM-MSCs (top) and SaOS-2 (bottom). The percentage of apoptotic SaOS-2 on PAM + Pyr increases from the control (5.4%): 5 min (6.7%), 10 min (10.5%) and 15 min (19.2%). Representative data at 24 hours are shown. Analysis were done in duplicates. (**B**) Representative images (left) and quantification (right) of immunofluorescence for γH2AX (Green) and DAPI (blue) after 2 h on (**B**) SaOS-2 and (**C**) hBM-MSCs treated with PAM +/− Pyr at 1 L/min and 10 mm for 10 min. Quantitative analysis are shown as percentage of γH2AX positive (green) vs the total number of DAPI (blue) positive nuclei at the indicated treatment times. PAM +/− Pyr on SaOS-2 induces no statistically significant differences exist at 10 (p value = 0.1573) and 15 min (p value = 0.008), showing only significant differences on 5 min treatment time (p value = 0.0008). PAM -Pyr is capable to affect DNA integrity on healthy cells (p value = 0.0002). Scale bar = 50 µM. Data are presented as mean, n = 3. Error bars represent the SD, and asterisks indicate statistically significant differences between the PAM +/− Pyr series (*p < 0.05; **p < 0.01, ***p < 0.001; two-sided Student’s t-test).

We found that PAM induced apoptosis (Annexin V positive cells) in SaOS-2 cells in a gas flow dependent manner in all the conditions studied, with only a marginal occurrence of other mechanisms of cell death such as necrosis (PI positive/Annexin V negative cells). Again, the induction of apoptosis is much more efficient in the absence of pyruvate reaching almost 90% of apoptotic cells after the treatment with plasma at 3 L/min, whereas the treatment in the presence of pyruvate produces approximately a 30% of apoptotic death after a 5 L/min treatment. Similarly, in experiments where cells were treated with PAM + Pyr treated with 1 L/min of gas flow for different times we found a selective time-dependent induction of apoptosis in SaOS-2 cells but not in hBM-MSCs (Fig. 4A).

In addition, PAM treatment is able to induce DNA damage as seen by the increase of cells presenting γH2AX foci (Fig. 4B). In line with our findings in survival and apoptotic assays, treatment with PAM-Pyr induces significantly higher levels of DNA damage than those achieved after the treatment with PAM + Pyr and the presence of pyruvate also determines that the genotoxic effect occurs specifically in OS cells and not in healthy cells (Fig. 4B,C). Altogether, PAM treatment efficiently induces apoptosis and DNA damage in OS cells and the presence or absence of pyruvate in its composition allows the modulation of its sensibility and selectivity.

### Plasma-generated hydrogen peroxide is scavenged by pyruvate and its levels correlate with PAM induced cytotoxicity

**Figure 5:**
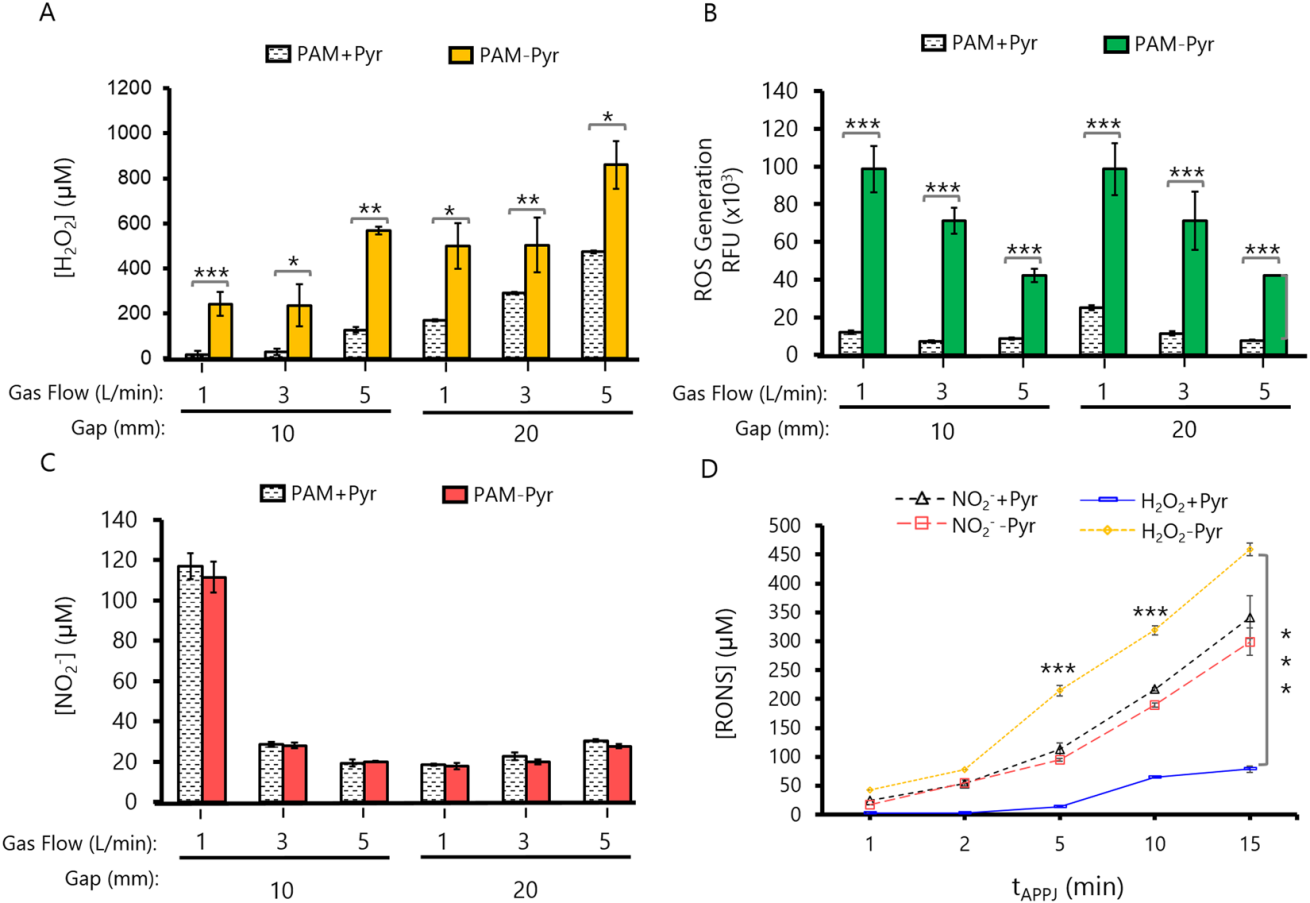
Profile of RONS on PAM depends on various APPJ operating parameters. Different physical parameters like the gap between APPJ nozzle to cell culture surface (10 or 20 mm), Helium Flow (1, 3, and 5 L/min) and treatment time (5 min) were used to obtain 2 mL of PAM +/− Pyr. (**A**) Micromolar (μM) concentration of Hydrogen Peroxide (H_2_O_2_) by AmplexRed/HRP assay. (**B**) ROS were measured by DCF-DA fluorescence intensity (x10^3^). (**C**) Micromolar (μM) concentration of Nitrites (NO_2_^−^) by Griess assay. (**D**) Concentration of NO_2_^−^ and H_2_O_2_ in PAM +/− Pyr at different treatment times (1 to 15 min at 1 L/min and 10 mm gap). PAM + Pyr not lead to significant differences on the concentration of NO_2_^−^, with a maximum of 341 ± 27 µM (PAM + Pyr) or 299 ± 10 µM (PAM-Pyr). Data are presented as mean, n = 3, Error bars represent the SD, and asterisks indicate statistically significant differences between the PAM +/− Pyr series (*p < 0.05; **p < 0.01; ***p < 0.001; two-sided Student’s t-test). Concentration of RONS was measured immediately after APPJ treatments using untreated DMEM as blank. The exact concentrations of RONS are presented in Table S2.

While the increasing gas flow modifies only slightly the amount of species detected in the gas phase (Fig. 1), important variations might occur during treatment of liquids regarding the diffusion of species, or convection of the liquid. This is clearly shown by the amount of reactive species formed in PAM at different gas flows (Fig. 5), which has been evaluated at two different distances from the tip of the jet.

It is known that plasma generates long-lived reactive species such as H_2_O_2_ or NO_2_^−^ (Fig. 5) in the treated liquids. Herein DMEM is evaluated, in the presence or absence of pyruvate. Pyruvate, is a component in some commercial formulations of cell culture media, and is also present naturally in the body. Its presence or absence in the PAM generates important differences with regard to the total reactive species and the hydrogen peroxide concentration. In PAM + Pyr we detected significantly lower concentrations of hydrogen peroxide, with differences ranging between 2 fold (5 L/min; 20 mm) and 15 fold (1 L/min; 10 mm) (Fig. 5A). In parallel, the amount of total ROS were up to 5 fold higher in PAM-Pyr (1 L/min; 10 mm) (Fig. 5B). The treatment distance does not significantly affect the level of total ROS generated in PAM-Pyr, which tends to decrease with the gas flow, and only at 1 L/min higher concentration is found at higher distance in PAM + Pyr. Opposite trend is observed in the concentration of H_2_O_2_, with concentration increasing with the gas flow and slightly higher amounts generated at longer distance both PAM +/− Pyr (Fig. 5A). The addition of this scavenger does not affect the concentration of NO_2_^−^ (Fig. 5C), which is found maximum at short distance and gas flow (1 L/min, 10 mm), while relatively low concentrations are generated in all other conditions evaluated. Micromolar concentrations of NO_2_^−^ and H_2_O_2_ are summarized on Table S2.

The concentration of H_2_O_2_ and of NO_2_^−^ was quantified in 2 mL of DMEM with/without pyruvate treated with plasma at 1 L/min from 1 min up to 15 min with a gap of 10 mm. Data confirm that the generation of RONS in the cell culture medium following plasma treatment is time dependent (Fig. 5D). The presence of pyruvate in the medium does not lead to significant differences on the concentration of NO_2_^−^. However, the concentration of H_2_O_2_ generated in PAM after the treatment with plasma is highly influenced by presence of pyruvate, being up to 100 times higher in the case PAM-Pyr media depending on the experimental conditions (Fig. 5D).

These results together with the cytotoxicity assays (Figs 2–4) show that CAP induced apoptosis in PAM-Pyr is related with an excess of lethal concentration of H_2_O_2_. Interestingly, the levels of NO_2_^−^ in PAM-Pyr not be correlated with the toxicity of the PAM.

### PAM-Pyr and H_2_O_2_ induce similar phosphorylation patterns in signaling kinases

**Figure 6:**
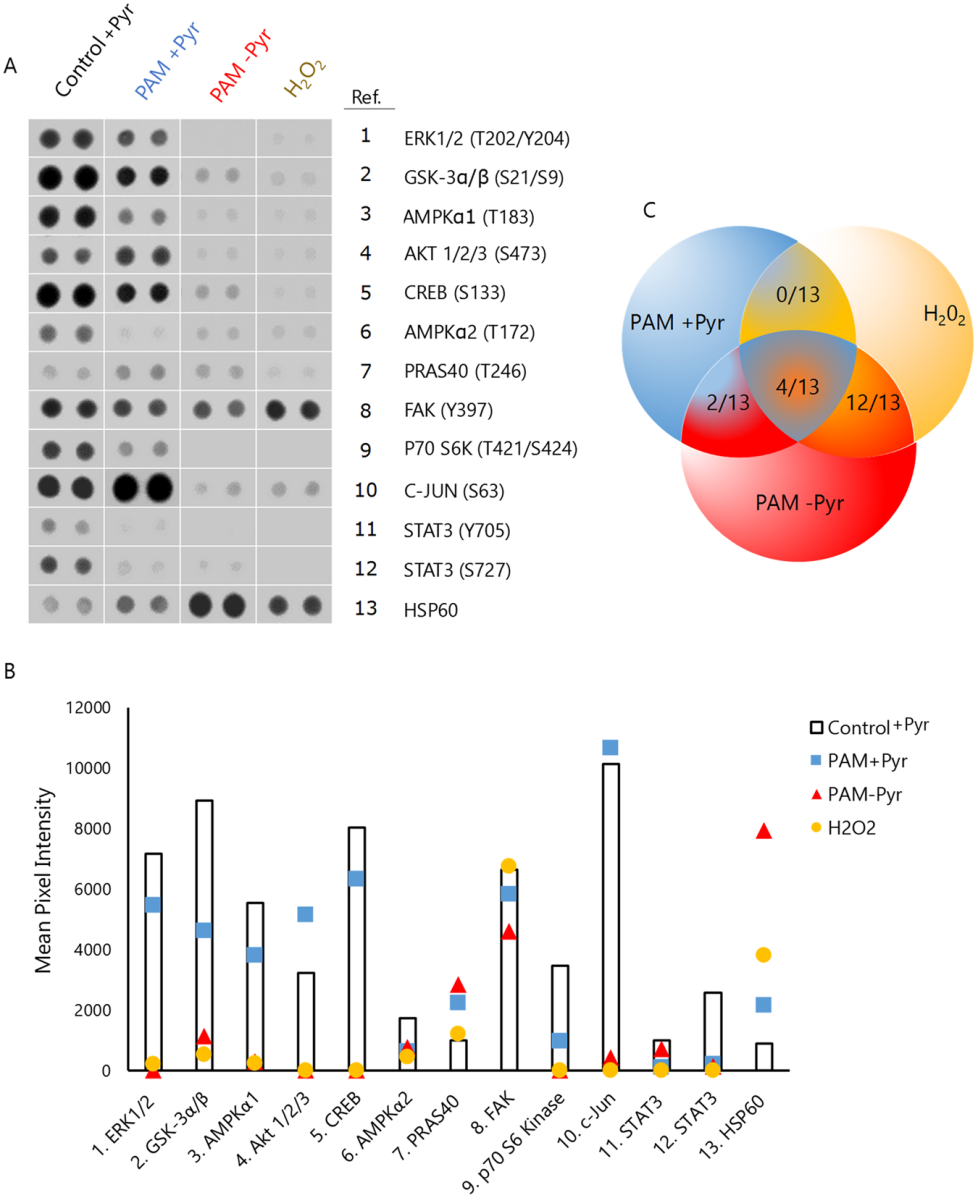
OS cells phosphorylation profiling affected by PAM. Phospho-kinase antibody array showing the phosphorylation/activation status of a panel of kinases in SaOS-2 cells after treatment with PAM +/− Pyr (10 mm, 1 L/min, 15 min) or H_2_O_2_ (400 μM) for 6 hours. (**A**) Representative spots and numbers listed correspond to 13/43 selected kinases. (**B**) Selected spots were digitized and mean pixel intensity is presented as columns (untreated) or colored markers (treatments). (**C**) Venn diagram illustrating the number of similar phosphorylation pattern between treatments PAM + Pyr (blue), PAM-Pyr (red) and H_2_O_2_ (yellow): PAM + Pyr & PAM-Pyr (2/13), PAM + Pyr & H_2_O_2_ (0/13), PAM-Pyr & H_2_O_2_ (12/13). Complete images of the phospho-kinase antibody array are presented as Fig. S2.

To obtain an in-depth view of the regulation of PAM-dependent cell signaling, a Proteome Profiler Human Phospho-kinase Array was employed to analyze the changes in the kinase phosphorylation profile of SaOS-2 cells after the treatment with PAM for 15 min. This treatment produced high NO_2_^−^ and low H_2_O_2_ concentrations in DMEM + Pyr and high concentration of H_2_O_2_ and NO_2_^−^ in DMEM - Pyr (Fig. 5D). Taking this into account, we used an equivalent dose of H_2_O_2_ to PAM-Pyr (400 μM) to compare the effects of PAM and H_2_O_2_ on the phosphorylation profile (Fig. 6).

This study reveals a differential regulation in 13 of the 43 kinases analyzed. On the one hand, the presence of Pyr in PAM produces decreased phosphorylation of ERK1/2 (T202/Y204), GSK-3α/β (S21/S9), AMPKα1 (T183), CREB (S133), AMPKα2 (T172), FAK (Y397), P70 S6K (T421/S424) and STAT3 (Y705/S727) and increased phosphorylation of AKT (S473), C-JUN (S63), and HSP60. On the other hand, PAM-Pyr induces a general inhibitory effect of most of kinases which is greater than that observed after PAM + Pyr treatment. Relevant differences associated to absence of pyruvate include the inhibition, rather than activation, of AKT and C-JUN and highly increased phosphorylation levels of HSP60. Notably, SaOS-2 cells treated with H_2_O_2_ closely mimic the kinase phosphorylation profile of PAM-Pyr treated cells (Fig. 6A,B). Thus, the phosphorylation levels of 12 of the 13 selected kinases were similarly increased or decreased in both treatments (Fig. 5C).

The similarity of the phosphorylation profiles induced by PAM-Pyr and H_2_O_2_ in relevant signaling kinases suggest that the increased levels of this ROS is a key mediator of the high cytotoxic effect induced by PAM in the absence of pyruvate and also that the differences observed after PAM-Pyr and PAM + Pyr treatments could be on the basis of the anti-tumor selectivity observed in the presence of pyruvate.

## Discussion

CAP has been suggested as a new therapy against cancer^41,51^, but its selectivity against cancer cells avoiding damage to healthy cells has been less investigated, as only around 30% of the published research reports selectivity. It has been described that it is possible to take advantage of the biological differences between cancer cells and normal cells to selectively kill the malignant cells. The disparities in ROS production and metabolism in cancer cells versus normal cells provides a biochemical basis to develop new therapeutic strategies to preferentially increase ROS to a toxic level in cancer cells. A “threshold concept” for cancer therapy has been proposed to explain the dual effects of oxygen radicals^52^. In cancer cells, if ROS levels reach the “threshold level” that overwhelms the antioxidant capacity, irreversible damage occurs and apoptosis is initiated, and as observed here, this threshold level is different for cancer and healthy cells.

In this work we employ an atmospheric pressure plasma jet (APPJ)^22^ to evaluate suitable treatment conditions to selectively damage SaOS-2 osteosarcoma cells, without affecting healthy hBM-MSCs. The first aim was to determine the relationship between the concentrations of plasma generated-RONS in PAM at different plasma treatment conditions with anti-osteosarcoma selectivity. This is intended to help answering the question of which of the plasma generated RONS is more relevant with regard the anti-tumoral selectivity of APPJ. To that aim, different parameters were modified during CAP treatment of DMEM: i. Distance to the liquid surface (10 or 20 mm); ii. Gas flow (1, 3, 5 L/min) and iii. Treatment time (1 to 15 min). DMEM cell culture medium with or without addition of sodium pyruvate was employed to investigate the specific role of H_2_O_2_ in the biological effects of PAM on osteosarcoma *in vitro*.

APPJ-treated PAM–Pyr was completely cytotoxic to SaOS-2 cells, decreasing hBM-MSC viability between 80 and 20% (Fig. 2A) and proliferation (Fig. 2B) in a gas flow dependent manner. This toxicity was partially abrogated in SaOS-2 in presence of pyruvate and even proliferation was stimulated in SaOS-2 (1 L/min, 10 mm) and hBM-MSC (Fig. 2B). Surprisingly, pyruvate effectively reduces PAM-induced apoptosis in OS (Fig. 3) and prevents apoptosis in healthy cells (Fig. 4A).

Due to the capacity of CAP to generate RONS, we evaluated its genotoxic potential by analyzing the levels of γH2AX. In our study, CAP-treated medium induced an increase in the level of γH2AX under all tested treatment conditions, with higher values after PAM-Pyr treatment (Fig. 4B,C). The decrease in γH2AX recorded after PAM + Pyr treatment suggests that H_2_O_2_ plays a pivotal role in the DNA damage induced by CAP. However, PAM + Pyr did not fully prevent the presence of γH2AX foci (Fig. 4B,C), so in line with previous works^32^ our results suggest that other plasma-generated RONS may be also activating DNA damage response. An important asset is that DNA damage is found preferably in SaOS-2 cells treated with PAM +/− Pyr (Fig. 4B,C). This, in conjunction with the fact that healthy cells do not enter in apoptosis (Fig. 4A), seems to indicate that PAM induces apoptosis and DNA damage mainly in osteosarcoma cells, but the absence of pyruvate increases the DNA damage, affecting both healthy and tumor cell lines (Fig. 4B,C). In hBM-MSCs, higher γH2AX levels after PAM-Pyr treatment (Fig. 4C) correlate with a decreased cell viability and proliferation (Fig. 2).

The involvement of RONS generated by plasmas in cell culture media in the anticancer effects of PAM has been reported in many studies, with special emphasis on the role of H_2_O_2_ and NO_2_^−^, among other species^36,40^. Many other studies suggest that H_2_O_2_ is the most relevant ROS from CAP in views of tumor cell killing^6,39,40,53^. Beckeschus *et al*. showed that the enzyme catalase very effectively suppressed the cytotoxic effects of PAM mediated by H_2_O_2_ ^54,55^. In this regard, our data show the same trend, the presence of scavengers such as Pyr or catalase, efficiently nullifies the cytotoxic potential of PAM (Fig. 3). In line with these data, it is reasonable to postulate that the sensitivity found in cells may be due to an excess of H_2_O_2_ when used in PAM-Pyr.

The gas phase of the plasma jet evaluated here contains several radical and ionic species (O*, *OH, N_2_^+^) (Fig. 1) which vary in concentration depending on treatment conditions such as the flow of the gas (He) employed to generate the discharge (Table S1). This and other parameters which modify the gas phase discharge, certainly influence on the amount of RONS formed in the cell culture medium due to plasma CAP treatment. Here, H_2_O_2_, NO_2_^−^ and total Reactive oxygen species (ROS) were quantified in PAM (Fig. 5). At short distance between the jet and the liquid surface, high NO_2_^−^ concentration is produced, deriving from NO_2_ dissolution from the plasma gas phase, especially at low gas flow^35^. As shown by Lu *et al*. [76] the amount of N in the effluent of a jet can be of 10 orders of magnitude higher at 10 mm than at 20 mm, coping well with these results.

Also, higher electron density may happen near the surface at shorter distances, leading to enhanced reactivity with the liquid; at higher gas flows increased water vapor mixing with the jet leads to higher H_2_O_2_ concentrations, etc. The basics of reactivity of plasmas with liquids have been summarized elsewhere^35^. The species measured in this work are an example of some of the many RONS that may be formed in CAP treated liquids. Other species that we could assume to be present in our PAM are NO_3_^−^ which is often quantified in saline solutions^56,57^, or cell culture media^42^, *OH that was observed in aqueous solution by electron paramagnetic resonance (EPR)^58^, or ONOO^−^ which is known also to be of importance in biological processes but not straightforward to measure in liquids.

In other works, NO_2_^−^ and H_2_O_2_ concentrations have shown a synergistic cytotoxic effect in PAM^40^. In our case, at 1 L/min we obtain the highest concentrations of nitrites (Fig. 5C), but an increase in the proliferation of hBM-MSCs and SaOS-2 is observed (Fig. 2A,B). In addition, PAM + Pyr activated at 1 L/min during 15 min (Fig. 4A) has less apoptotic potential than a 5 min-treatment with PAM-Pyr (Fig. 3). Furthermore, pyruvate significantly reduces the concentration of H_2_O_2_ (Fig. 5D). Therefore, our data reveal that PAM-Pyr induced apoptosis in OS cells is mainly related to H_2_O_2_ concentration. Thus, in line with other works^36,38,40^, SaOS-2 cells display APPJ-treatment time dependent apoptosis (Fig. 4A). However, the fact that PAM + Pyr with low H_2_O_2_ concentration can induce apoptosis in a gas flow (Fig. 3) and treatment time dependent manner (Fig. 4A), suggest that other RONS formed in PAM and not studied in this work could be acting on plasma induced cell toxicity. In any case, in PAM-Pyr we obtained enough H_2_O_2_ concentration to induce apoptosis (Fig. 5A–D & Table S2).

Our results reflect on the important effect of sodium pyruvate, which abolishes the cytotoxic potential of PAM by increasing cell proliferation and reducing the levels of apoptosis induced by PAM on osteosarcoma cells with respect to PAM-Pyr. Furthermore, absence of Pyruvate increases the efficacy of PAM in cancer and healthy cells, eliminating the anti-tumoral selectivity. This can be directly related to high H_2_O_2_ concentrations. These results can be explained considering two aspects of pyruvate: i. Its scavenging properties as it reacts with H_2_O_2_ to produce CO_2_, H_2_O and acetate through an oxidative decarboxylation^59,60^, ii. Its biological effects: Pyr protects cells from H_2_O_2_ cytotoxicity by mitochondrial regulation, especially if the ROS are generated in cell culture medium^61–63^, and iii. Pyr protects cells by activating Akt signaling pathway and increasing defenses to H_2_O_2_ like glutathione peroxidase activity^64^. To this regard, many studies defend that CAP produces its cytotoxic effects through a reduction of antioxidant defenses such as glutathione peroxidase^27^, or through depolarization of the mitochondrial membrane^5,25^. Thus, it is clear that pyruvate is a relevant player to take into account to address CAP efficiency.

We have shown that cytotoxic effects of PAM in our experimental setting are related mainly to the concentration of H_2_O_2_. This is further confirmed by the fact that PAM-Pyr generates a phosphorylation profile very similar to that obtained after treatment with H_2_O_2_ (Fig. 6). The less effective treatment in inducing apoptosis (PAM + Pyr) produces signals related to sublethal levels of oxidative stress and proliferation such as c-JUN^65^ and AKT^64^. It should be underlined that treatment with PAM (+/− Pyr) as well as with H_2_O_2_, affects proteins related to mitochondrial stress and apoptosis like HSP60^66^, or STAT3 pathway^67^. This is in line with previous results relating the cytotoxic activity of PAM with an increase in mitochondrial damage^6,25,47,68^. These three treatments affect cell signaling related to autophagy like STAT3 and AMPKα1/AMPKα2. Furthermore AMPK inhibition increases chemo-sensitivity^69^ and STAT3 downregulation suppresses osteosarcoma cell growth and induces apoptosis^70^. Furthermore, only treatment with PAM (+/− Pyr) affects FAK activation, a kinase associated to cell invasion and poor prognosis in sarcoma^71^. All this confirms that PAM may have therapeutic potential in the treatment of osteosarcoma.

While other studies have described that the anti-tumor selectivity of CAP depends on the activation of p53 and ERK^47,68^, our data in SaOS-2 cells (*P53 null*)^72^ indicate that p53 (S46) is not related to PAM cytotoxicity (Fig. S2), while ERK is downregulated (Fig. 6). In general, H_2_O_2_ affects the same molecular targets than PAM-Pyr in a total of 12/13 of the altered kinases. The signaling affected by PAM seems to depend on the concentration of reactive species and may be related to an adaptive response to oxidative stress, although a more thorough study would be recommended.

Our data show that PAM-Pyr has greater cytotoxic effect accompanied with a loss of anti-tumor selectivity. This is a clear indication that the selectivity of PAM *in vitro* is H_2_O_2_-dependent. In the clinical setting, other liquid media such as saline solutions are expected to be employed to deliver RONS from plasmas rather than PAM, so different concentrations of RONS may be expected. Nevertheless, this work is relevant because Pyr is found in the tumoral environment, and as described here, it may enormously modify the cytotoxic effect of plasma-activated saline solutions when injected *in vivo*. It is therefore necessary to take into account all components in the cancer environment to propose relevant *in vitro* studies and obtain a correct *in vivo* plasma application targeting only cancer cells.

While we have shown that concentration of H_2_O_2_ has a high impact on selectivity, there are many studies describing the anti-carcinogenic effects of CAP by many other reactive species such as O_2_^−^, OH^*^, NO, O, NO_3_^−^, NO_2_^−^ and ONOO^−^ ^73^. However, when using PAM or saline solutions activated by plasma^35,74^, usually only H_2_O_2_ ^41,48,75,76^ or NO_2_^−^ ^38,40^ are determined due to their long life and easier detection methods available. As mentioned above for the case of Pyr, in the clinical application, RONS generated by plasma may not be present in same concentrations as those determined in liquids such as PAM because other factors can interfere with the formation and stability of RONS e.g. proteins, organic and inorganic molecules etc.

Therefore, it can be inferred that to investigate the antitumor selectivity of CAP *in vitro*, PAM should be obtained in conditions leading to an “equilibrated” cocktail of RONS, and not only an excess of H_2_O_2_.

## Methods

### Plasma jet device

An atmospheric pressure plasma jet (APPJ) was created using Helium (5.0 Linde, Spain) as plasma gas in a jet design with a single electrode as described elsewhere^77^. The electrode was connected to a commercial high voltage power supply from Conrad Electronics (nominally 6 W power consumption). The discharge was operating with sinusoidal waveform at 25 kHz with (U) ~ 2 kV and (I) ~ 3 mA. He flow in the capillary was regulated between 1 and 5 L/min through a MassView flow controller (Bronkhorst, Netherlands).

### Optical emission spectroscopy (OES)

OES was used to determine the main plasma emitting species. The equipment used was a spectrometer F600-UVVIS (StellarNet, Tampa, FL, USA), which was connected to an optical fiber with lens that collected information from the measure point near the plasma jet. For data processing the SpectraWiz software (StellarNet, Tampa, FL, USA) was used. The optical fibre was placed perpendicular to the jet and measurements were made about ten millimetres below the beginning of the jet (in the post-discharge, d = 10 mm). All results were obtained with an integration time of 1000 ms and an average of 10 scans.

### Plasma activated medium (PAM)

We studied the concentration of RONS and cellular responses generated after APPJ treatments on 2 mL DMEM, high glucose, no glutamine, no phenol red (Gibco^TM^, cat.no 31053028, Carlsbad, CA, USA) with or without 0.1 g/L Sodium Pyruvate (Gibco^TM^, cat.no 11360070, Carlsbad, CA, USA) in a 24-well plate. Catalase (MP Biomedicals™, cat.no 9001-05-2) was added at 20 μg/mL immediately after (within 5 s) after plasma treatments, prior to putting it in contact with cells.

Three APPJ parameters were varied: 1. He flow rate: 1, 3 and 5 L/min; 2. Gap between APPJ nozzle and DMEM Surface: 10 or 20 mm; 3. Treatment time: between 1 and 15 min.

### Concentration of RONS in PAM

#### Nitrites

Determination of NO_2_^−^ concentration in PAM was performed using Griess reagent^78^. The Griess working solution used was obtained by dissolving 1% wt/v of sulphanilamide, 0.1% wt/v of NEED and 5% w/v of phosphoric acid in de-ionized water. 50 μL of Griess solution were added on 50 μL of sample in 96 well-plates. The plates were incubated for 10 min at room temperature protected from light. The absorbance was measured at λ_abs_ = 540 nm using a Synergy HTX Hybrid Multi Mode Microplate Reader (BioTek Instruments, Winooski, Vermont, USA). The [NO_2_^−^] in each sample was determined from the absorbance values by using a calibration curve made from a commercial standard Sodium Nitrite 14 mM (CellBiolabs, cat.no 280201, San Diego, CA, USA) diluted on DMEM.

#### Hydrogen peroxide

The concentration of hydrogen peroxide was determined by Amplex™ Red Hydrogen/HRP Peroxide Kit (cat.no A2218, Invitrogen, Carlsbad, CA, USA) following the manufacturer’s protocol. Since the higher concentration of H_2_O_2_ able to be processed properly by this reagent is around 5 µM of H_2_O_2_, PAM with sodium pyruvate were diluted 10 and PAM without sodium pyruvate were diluted 10–100 times previously to the addition of the reagent. In this case, for hydrogen peroxide detection, 50 μL of the Amplex^TM^ Red/Horseradish Peroxidase (HRP) reagent were added to 50 μL of PAM in a dark 96-well plate and incubated for 30 min at room temperature. Subsequent fluorescence measurements were performed by means of a Synergy HTX Hybrid Multi Mode Microplate Reader (BioTek Instruments, Winooski, Vermont, USA), with fluorescence filters centred at λ_ex_ = 560/20 nm and λ_em_ = 590/20 nm as excitation and emission wavelengths, respectively. Concentrations of H_2_O_2_ in PAM generated by plasma treatment were obtained from fluorescence values using a calibration curve following the manufacturer’s protocol.

#### Generation of total ROS

Total ROS were determinated by a fluorescence method using 2′,7′ Dichlorodihydr- ofluorescein diacetate (DCFH-DA), used as scavenger of ROS in liquid media (Sigma-Aldrich, cat.no D6883, Sant Luois, MO, USA). DCFH was added to 150 μL PAM in proportion 1 μL of 2 mM 2,7-DCHF for of PAM in a dark 96-well plate. After 30-min incubation at room temperature, fluorescence intensity was read with a Synergy HTX Hybrid Multi Mode Microplate Reader using λ_ex_ = 485/20 nm and λ_em_ = 528/20 nm as excitation and emission wavelength filters, respectively.

#### Cell culture

We evaluated the effects of PAM on the osteosarcoma cell line SaOS-2 and healthy Human Bone Marrow Mesenchymal Stem Cells (hBM-MSCs) (both cell types obtained from ATCC, USA). In this study cell lines were grown in Dulbecco’s Modified Eagle Medium (DMEM) with glucose (4,5 g/L), pyruvate, no glutamine (Gibco™ cat no. 21969035, Carlsbad, CA, USA), to which we add 10% fetal bovine serum (FBS) (Gibco™ cat no. 10270098, Carlsbad, CA, USA), 2mM L-glutamine (Gibco™, cat.no 25030081), 100 units/mL penicillin (Gibco™, cat.no 15140122) and 100 µg/mL streptomycin (Gibco™, cat.no 15140163). The cells were incubated at 37 °C, 95% humidity and 5% CO_2_.

#### Cell viability assay

To evaluate the antitumor effects of PAM treatment, a WST-1 (Roche, cat.no 05015944001, Mannheim, Germany) cell proliferation assay was performed according to the manufacturer’s instructions. Cells were seeded in a 96-well plate at a density of 5 × 10^3^ per 1000 μL of culture medium. On the following day, the culture medium was replaced with 150 μL of PAM. After 24 hours, WST-1 working solution (18 μL/mL) was added to each well and plates were incubated at 37 °C for 60 min. Absorbance was measured at λ_abs_ = 440 nm. Each experiment was performed by independent triplicates. Cells untreated by PAM were used as control.

#### Proliferation analysis

To evaluate long-time PAM effects on proliferation ability we used the xCELLigence system (ACEA Biosciences, Inc, San Diego, CA, USA)^79^. Cells were seeded in specially designed microtiter plates containing interdigitated gold microelectrodes at a density of 10 × 10^3^ per 500 μL of culture medium. On the following day, 400 μL of culture medium was replaced with 400 μL of PAM. The continuous cell impedance noninvasive monitoring by the xCELLigence system was measured every hour until the end of experiment (70 h post-treatment).

#### Analysis of apoptosis/necrosis activation

Cells were seeded on 6-well plate at a density of 50 × 10^3^ per 1000 μL of culture medium, on the following day, the culture medium was replaced with 1500 μL of PAM. After PAM treatment on indicated conditions, cells were stained with Dead Cell Apoptosis Kit with Annexin V Alexa Fluor™ 488 & Propidium Iodide (PI) (Invitrogen, cat.no 10257392, Carlsbad, CA, USA) following the manufacturer’s protocol. Cell counts were determined by flow cytometry, and data analysis was performed with FlowJo Software (https://www.flowjo.com/).

#### DNA damage

Cells were seeded on 8-well chamber slides at a density of 15 × 10^3^ per 500 μL of culture medium, on the following day, the culture medium was replaced with 400 μL of PAM. After PAM treatment on the indicated conditions cells were fixed with 4% paraformaldehyde in PBS for 15 min, washed three times with PBS, and permeabilized with 0.1% Triton X-100 on ice for 5 min. They were washed three times with PBS and blocked with SuperBlock™ (TBS) (ThermoScientific, cat.no 37535, Carlsbad, CA, USA) during 1 h. Cells were stained overnight at 4 °C on rocking platform for Anti-phospho-Histone γH2AX (Ser139) mouse Antibody, clone JBW301 (Merk Millipore, cat no. 05-636, Burlington, MS, USA) using a dilution 1:500. Following staining, cells were washed three times with PBS and incubated for 1 h at room temperature with Goat Anti-Mouse IgG H&L (Alexa Fluor® 488) (Abcam, ab150113, Cambrigde, UK). Samples were washed three times with PBS, and mounted using ProLong® Gold antifade with DAPI (LifeTechnologies, cat no. P36931, Carlsbad, CA, USA), be imaged with a Zeiss laser scanning microscope. Immunofluorescence images were taken at 25x.

#### Human proteome profiler array

The phosphorylation profile was analyzed in the PAM treated cells using the Proteome Profiler Human Phospho-Kinase Array (R&D Systems, cat.no ARY003B, Minneapolis, MN, United States). Cells (1 × 10^6^) were plated in a 100-mm dish, on the following day, the culture medium was replaced with 8000 μL of the indicated treatments (Fig. 6). 6 h post-treatment cells were collected by scraping. 300 μg total protein were applied per array set comprised of two nitrocellulose membranes with the spotted capture antibodies. The bound material was detected using the biotinylated antibodies followed by streptavidin conjugated with HRP. Chemiluminiscence was detected using Odyssey Fc imaging system and the software Image Studio from LI-COR (Lincoln, NE, USA). The pixel density of the background was substracted from the signal of each spot, and the average of duplicate spots was determined using the ImageJ software.

### Statistical analysis

All data are presented as means ± SD. Statistical analysis of the data was performed using a Student’s t-test. p-values < 0.05 were considered statistically significant.

## Conclusion

Overall, this work shows that the reactive species generated by CAP determine the selective anti-tumoral potential of PAM. The concentrations of RONS depend on the plasma process parameters and largely on culture media formulation. Our results reveal that sodium pyruvate plays a pivotal role on cold atmospheric plasma application in cancer treatment; On the one hand pyruvate diminish the cytotoxic potential of PAM both in cancer and especially healthy cells, thus enabling the possibility of finding a therapeutic window and selectively eliminating cancer cells. On the other hand, absence of pyruvate increases effects of PAM through a H_2_O_2_ dependent mechanism in cancer and non-cancer cells, resulting in a loss of anti-tumor selectivity. Regardless of pyruvate, PAM produces inhibition of relevant kinases such as AMPK or STAT3, thus revealing itself as a potential therapy against osteosarcoma.

## Supplementary information

Supplementary Information
