# Supplementary material for: Pyruvate Plays a Main Role in the Antitumoral Selectivity of Cold Atmospheric Plasma in Osteosarcoma
Source: Sci Rep. 2019 Jul 23;9:10681. doi: 10.1038/s41598-019-47128-1 (PMC6650457; doi:10.1038/s41598-019-47128-1)
Supplement: Supplementary file 1 — Supplementary Information [file 41598_2019_47128_MOESM1_ESM.docx]

**SUPPLEMENTARY INFORMATION**

PYRUVATE PLAYS A MAIN ROLE IN THE ANTITUMORAL SELECTIVITY OF COLD ATMOSPHERIC PLASMA IN OSTEOSARCOMA

Juan Tornin^1,2^, Miguel Mateu-Sanz^1,2^, Aida Rodriguez^3^, Cédric Labay^1,2^, Rene Rodríguez^3,4,5^, and Cristina Canal^1,2,§^

^
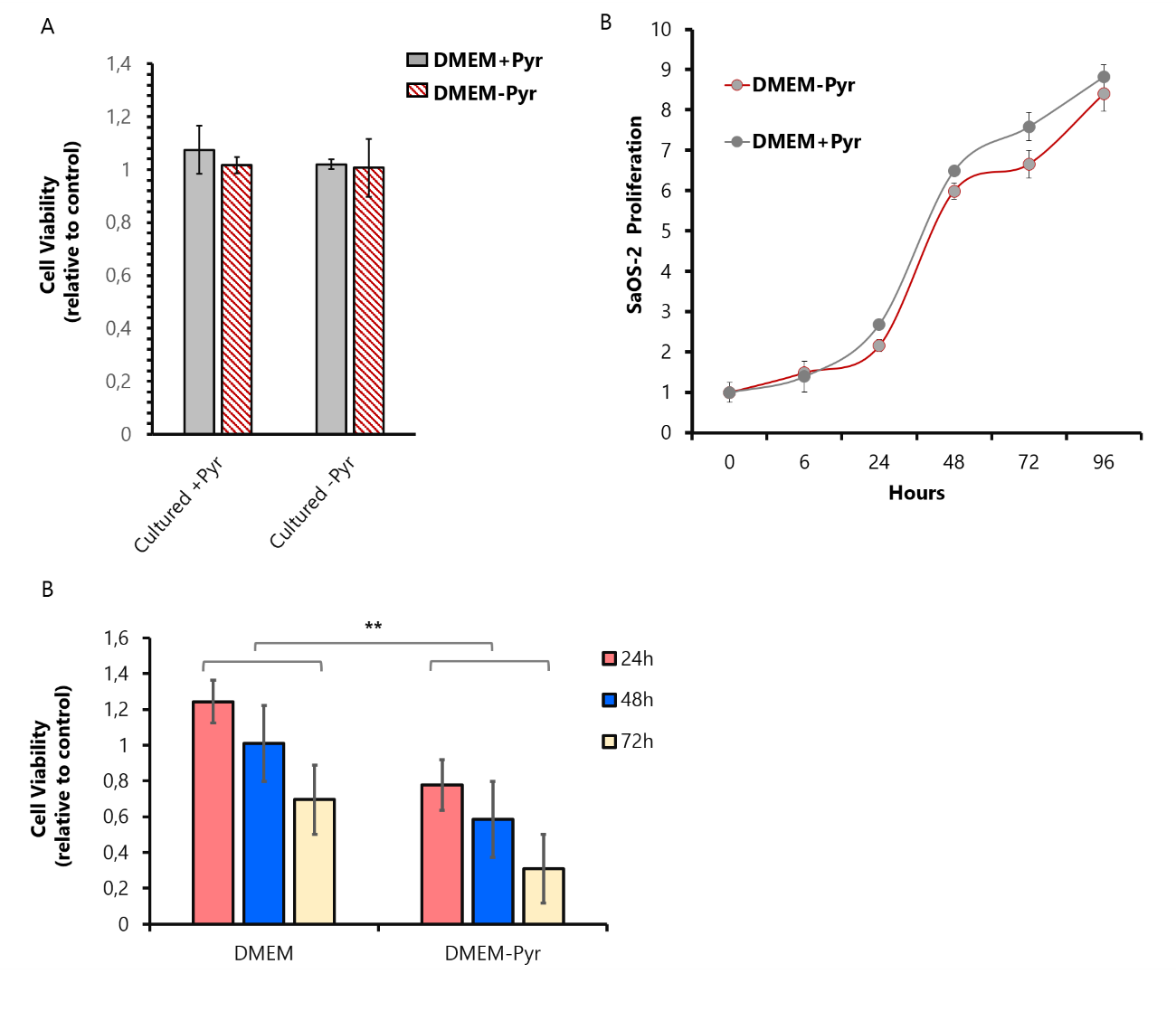
^

C

**Figure S1.** Cytotoxicity of PAM is not related to lack or shock of Pyr. (A) Effects of DMEM+/-Pyr on SaOS-2 viability were detected by WST-1 assay, measured 24h after exposure. Cell viability is expressed relative to the corresponding control cells cultured in DMEM+/-Pyr. Data show that untreated controls are not affected by the cell culture media in which cells are grown prior to the assay. (B) Proliferation curves of SaOS-2 cells up to 96h. 500 cell/well were seeded on 96 well plates, and cultured in DMEM+Pyr or –Pyr. The proliferation of SaOS-2 cells is unaffected by Pyr. (C) Effects of PAM+Pyr (activated by plasma during 5 minutes at 1L/min helium flow and 10mm gap) on cell viability were detected by WST-1 assay on SaOS-2, measured 24, 48 and 72h after exposure. Data are presented as mean, n=3. Error bars represent the SD, and asterisks indicate statistically significant differences between the cell cultured in DMEM+/-Pyr series (** p < 0.01; One-way ANOVA test).

**
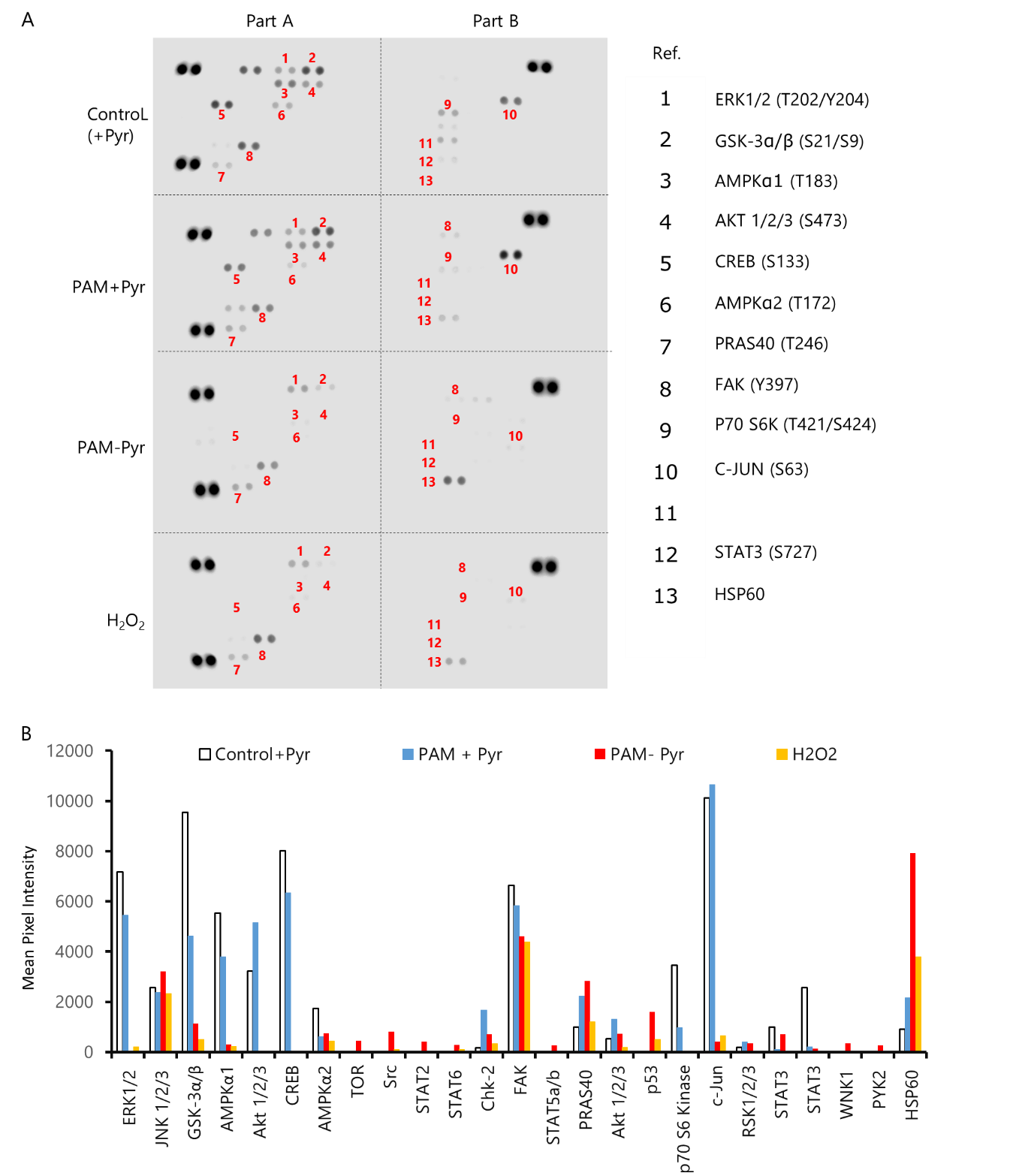
**

**Figure S2. Proteome Profiler Human Proteome Array**. (A) Complete image of the phospho-kinase antibody array. (B) Complete quantification of 43 kinases analyzed by pixel intensity. Only kinases yielding signal are represented.

**Supplementary Table 1. Optical Emission Spectra intensity of different species in the plasma gas phase**. Data show intensity (in arbitrary units) of the indicated ions, metastables and radicals at the gas flow employed in this work (1, 3 or 5L/min).

| Gas flow (L/min) | N_2_ 2^nd^+  (337 nm) | *OH  (316 nm) | N_2_^+^ 1^st^-  (380 nm) | He  (706 nm) | *O  (777nm) |
| --- | --- | --- | --- | --- | --- |
| **1** | 1.00x10^7^ | 4.69x10^6^ | 1.85x10^6^ | 0 | 0 |
| **3** | 1.19x10^7^ | 5.35x10^6^ | 2.42x10^6^ | 1.64x10^6^ | 4.70x10^5^ |
| **5** | 1.09x10^7^ | 4.93x10^6^ | 2.27x10^6^ | 1.97x10^6^ | 1.92x10^6^ |

**Supplementary Table 2. Concentrations of RONS in the Plasma Activated Medium with or without Pyruvate.** Concentration of NO_2_^-^ and H_2_O_2_ on PAM+/- Pyr treated with plasma for 5 min at 10 or 20 mm (gap). Green colour indicates low, yellow medium and red high micromolar concentrations or both RONS. Untreated DMEM was used as blank.

| Gap | Gas Flow (L/min) | [NO_2_^-^] (μM) | | [H_2_O_2_] (μM) | | |
| --- | --- | --- | --- | --- | --- | --- |
|  |  | PAM+Pyr | PAM- Pyr | PAM+ Pyr | PAM- Pyr | |
| 10mm | 1 | 116 ± 6 | 111 ± 7 | 16 ± 14 | | 241 ± 52 |
|  | 3 | 28 ± 1 | 27 ± 1 | 27 ± 14 | | 234 ± 93 |
|  | 5 | 19 ± 1 | 19 ± 0.2 | 125 ± 12 | | 567 ± 16 |
| 20mm | 1 | 18 ± 0.5 | 17 ± 1 | 168 ± 3 | | 499 ± 101 |
|  | 3 | 22 ± 1 | 19 ± 1 | 290 ± 5 | | 503 ± 120 |
|  | 5 | 30 ± 0.8 | 27 ± 1 | 472 ± 5 | | 859 ± 105 |
